# Modulation of the Superconducting Phase Transition in Multilayer 2H-NbSe_2_ Induced by Uniform Biaxial Compressive Strain

**DOI:** 10.1021/acs.nanolett.4c02421

**Published:** 2024-07-30

**Authors:** Eudomar Henríquez-Guerra, Lisa Almonte, Hao Li, Daniel Elvira, M. Reyes Calvo, Andres Castellanos-Gomez

**Affiliations:** † Departamento de Física Aplicada, Universidad de Alicante, 03690 Alicante, Spain; ‡ Instituto Universitario de Materiales IUMA, Universidad de Alicante, 03690 Alicante, Spain; § BCMaterials, Basque Center for Materials, Applications and Nanostructures, 48940 Leioa, Spain; ∥ 2D Foundry Group, Instituto de Ciencia de Materiales de Madrid, Consejo Superior de Investigaciones Científicas, 28049 Madrid, Spain; ⊥ IKERBASQUE, Basque Foundation for Science, Plaza Euskadi 5, 48009 Bilbao, Spain

**Keywords:** 2D materials, superconductivity, strain engineering, phase transition, niobium diselenide, biaxial compressive strain

## Abstract

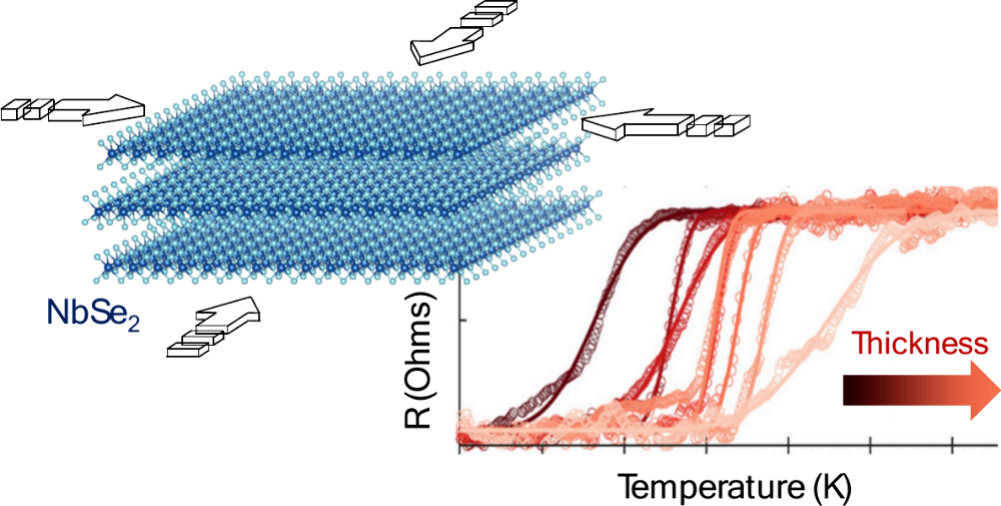

Strain is a powerful tool for tuning the properties of two-dimensional materials. Here, we investigated the effects of large, uniform biaxial compressive strain on the superconducting phase transition of multilayered 2H-NbSe_2_ flakes. We observed a consistent decrease in the critical temperature of NbSe_2_ flakes induced by the large thermal compression of a polymeric substrate (>1.2%) at cryogenic temperatures. For thin flakes (∼10 nm thick), a strong modulation of the critical temperature up to 1.5 K is observed, which monotonically decreases with increasing flake thickness. The effects of biaxial compressive strain remain significant even for relatively thick samples up to 80 nm thick, indicating efficient transfer of strain not only from the substrate to the flakes but also across several van der Waals layers. This work demonstrates that compressive strain induced from substrate thermal deformation can effectively tune phase transitions at low temperatures in 2D materials.

The phenomenon of superconductivity, characterized by its abrupt transition to zero resistance and expulsion of magnetic fields below a certain temperature, has long captivated physicists worldwide. This intriguing behavior, first observed more than a century ago,^1^ continues to fascinate fundamental researchers and holds promise for transformative applications in various fields.^2^

In recent decades, researchers have explored diverse avenues to manipulate this phase transition and in particular its critical temperature *T*_c_, below which materials enter the superconducting state. Techniques ranging from light irradiation^3,4^ to electrostatic doping^5,6^ have been employed in efforts to modulate *T*_c_. Notably, pressure and strain represent particularly powerful tools in this pursuit, offering direct control over material band structure and electron correlation effects governing superconductivity.^7,8^

Among the materials investigated, niobium diselenide (NbSe_2_) has emerged as a promising material for exploring superconductivity because of the possibility of getting ultraclean, freshly cleaved and dangling bond free surfaces by mechanical exfoliation. Mechanical exfoliation also offers the possibility to isolate very thin layers of NbSe_2_, even reaching down to single-unit cell thickness,^9^ which opens the door to study superconductivity at the true 2D limit^10,11^ and, having a high surface-to-volume ratio, allows to effectively tune the properties of NbSe_2_ nanosheets with external stimuli.^12^ Among the different tuning knobs, hydrostatic pressure^13−15^ and strain^16,17^ have also been investigated. These previous studies have primarily focused on the effects of uniaxial strain and hydrostatic pressure on bulk NbSe_2_, revealing intriguing modifications in *T*_c_ and electronic structure. While applying hydrostatic pressure up to 1.4 GPa has led to a 0.6 K increase in the transition temperature of bulk NbSe_2_, piezoelectric-actuated uniaxial strain of up to ±0.7% (compressive and tensile) resulted in a decrease in the transition temperature by up to 1.8 K for both types of strain. However, investigations into uniform biaxial strain effects in thin flakes of layered superconductors, and specifically of NbSe_2_, remain limited,^18^ despite their potential for more efficient strain transfer and enhanced tunability.

In this work, we investigated the effect of large and homogeneous biaxial compressive strain (>1.2%) on the superconducting phase transition of 2H-NbSe_2_ thin flakes deposited onto polycarbonate (PC) substrates with prepatterned electrodes. During the cooling process from 300 to 4.3 K, biaxial compressive strain is transferred to the NbSe_2_ flakes due to the thermal compression of the PC substrate. By measuring the two-terminal resistance of several NbSe_2_ devices on PC substrates, we observed a consistent decrease in the superconducting critical temperature as compared to similar devices prepared on SiO_2_/Si substrates (substrates inducing negligible strain). Our findings demonstrate a substantial modulation of the superconducting phase transition induced by biaxial compressive strain. The effect is more pronounced for thinner flakes (10 nm), with changes in *T*_C_ of up to 1.5 K, but remains noticeable for flakes as thick as 86 nm. These results demonstrate not only a significant effect of compressive strain in the superconducting properties of NbSe_2_ thin flakes, but also indicate an efficient strain transfer across a large number of van der Waals layers. Our findings not only contribute to the fundamental understanding of superconductivity in two-dimensional materials but also pave the way for practical applications harnessing strain as a versatile tool for tailoring material functionalities.

Thin 2H-NbSe_2_ (hereafter NbSe_2_) flakes were mechanically exfoliated and deposited onto a transparent viscoelastic membrane, where they were examined by optical microscopy in transmission mode to estimate their thickness. From this inspection and relative transmittance measurements, pairs of flakes with similar thickness were selected (see Supporting Information, Section S1). Subsequently, one of the two flakes was placed onto a polycarbonate (PC) substrate, while the other flake was deposited onto a SiO_2_/Si substrate, in both cases via an all-dry transfer method (see ref.^19^ and the Materials and Methods section). Electrodes separated by an ∼10 μm gap had been prelithographed on both substrates prior to flake deposition. SiO_2_/Si-based and PC-based NbSe_2_ devices are schematically depicted in Figure 1a and 1b, respectively. The large thermal expansion coefficient and Young’s modulus of polycarbonate makes it a suitable material for strain-transfer experiments with 2D materials.^20,21^ Recently, the thermal compression and efficient strain transfer onto single-layer transition metal dichalcogenides (TMDs) has been quantified in a very low-temperature regime,^22^ making these substrates an interesting platform for studying the effects of large compressive strain in 2D materials at low temperatures. In fact, during the elaboration of this manuscript, KP Yip et al. used a similar platform to subject a bulk (∼10 μm thick) layered superconductor, MoTe_2_, to homogeneous biaxial strain.^23^

**Figure 1:**
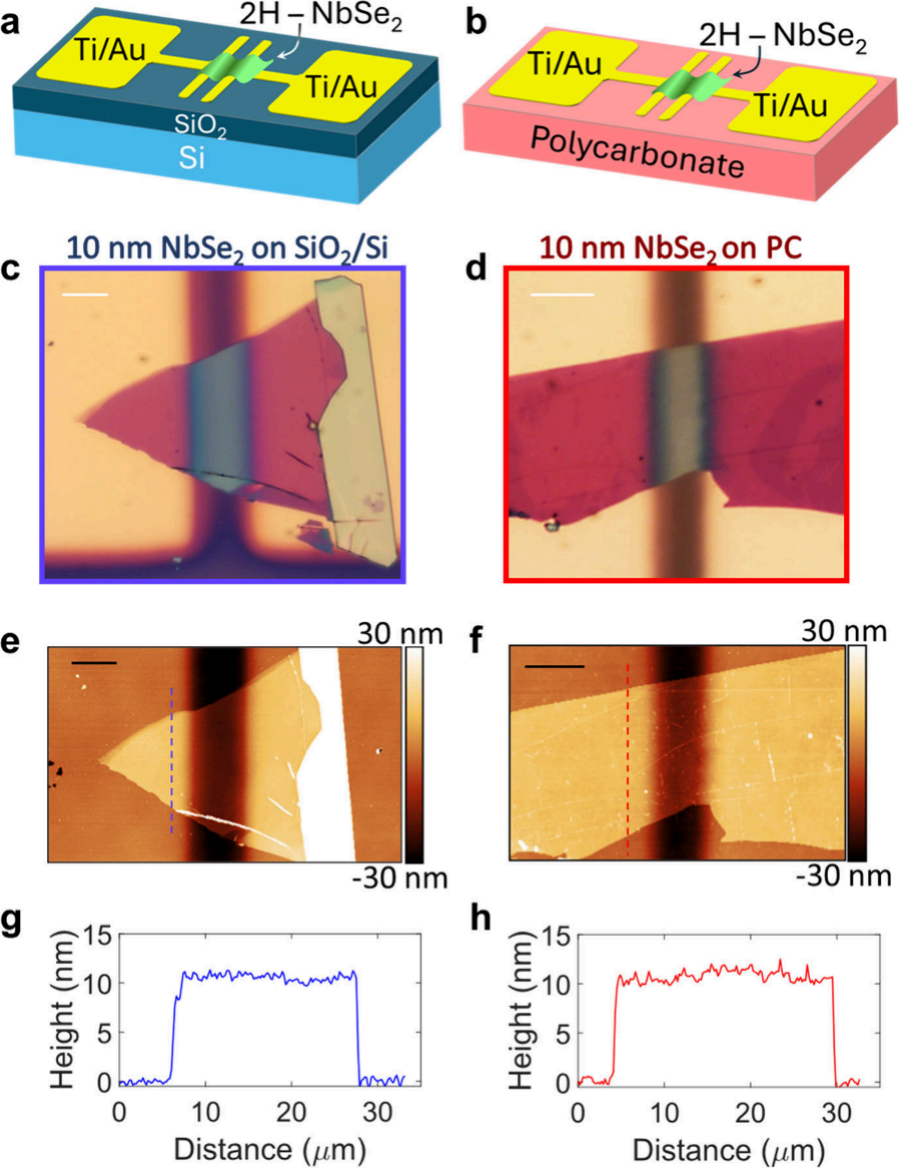
Scheme of the NbSe_2_ devices prepared on (a) SiO_2_/Si and (b) polycarbonate (PC) substrates. Optical image in reflection mode of a NbSe_2_ flake transferred onto (c) SiO_2_/Si and (d) onto a polycarbonate (PC) substrate with prepatterned contacts. White scale bars represent 10 μm. (e, f) AFM topographic image of the devices shown in panels (c) and (d), respectively. The AFM topography was acquired after completing all transport measurements. The black scale bar indicates 10 μm. (g, h) Height profile along the indicated path (dashed line) in (e) and (f), respectively. From the profiles, a thickness of 10 ± 2 nm is extracted for both devices.

Figure 1c,d presents optical images in reflection mode of two NbSe_2_ devices, each ∼10 nm thick, prepared on SiO_2_/Si and PC substrates, respectively. The thickness of these devices was confirmed using atomic force microscopy (AFM) topography measurements (Figure 1e-f). Height profiles confirm a thickness of 10 ± 2 nm for both devices on SiO_2_/Si, and on PC, respectively (Figure 1g-h, see Materials and Methods). The superconducting phase transition of NbSe_2_ has been shown to vary with flake thickness for samples below 8 nm.^24−26^ Therefore, we chose to study NbSe_2_ devices with thickness greater than 10 nm, so that their phase transition temperature was close to that of the bulk.

Next, two-terminal resistance was measured as a function of temperature for both 10 nm NbSe_2_ devices on PC and SiO_2_/Si (Figure 2a). Resistance was measured using an AC lock-in technique in a constant current configuration (see Materials and Methods section) while temperature was ramped down from 300 to 4.3 K at an approximate rate of 1 K/min. In Figure 2a, the residual resistance (*R*_contact_), determined as the resistance at base temperature (4.3 K), has been subtracted for the sake of comparison. For both devices, resistance decreases similarly with lowering temperature from 300 K to base temperature (Figure 2a). However, a zoom into the temperature range 5–8 K (in Figure 2b) reveals remarkable differences between the evolution of resistance with temperature for each device. In both cases, a resistance drop appears below a certain critical temperature, indicating the transition of the material from the normal metal to the superconducting state.^24^ For the device on SiO_2_/Si, where a negligible thermal-compression induced strain is expected, an abrupt drop of resistance is observed around ∼7 K. This is the critical temperature (*T*_c_) value expected for bulk samples^13^ and, in general, for flakes composed of more than 13 layers.^24,27,28^ In contrast, for the NbSe_2_ device on PC the resistance drops over a broader temperature range, centered around a significantly lower temperature of ∼5 K (see Figure 2b).

**Figure 2:**
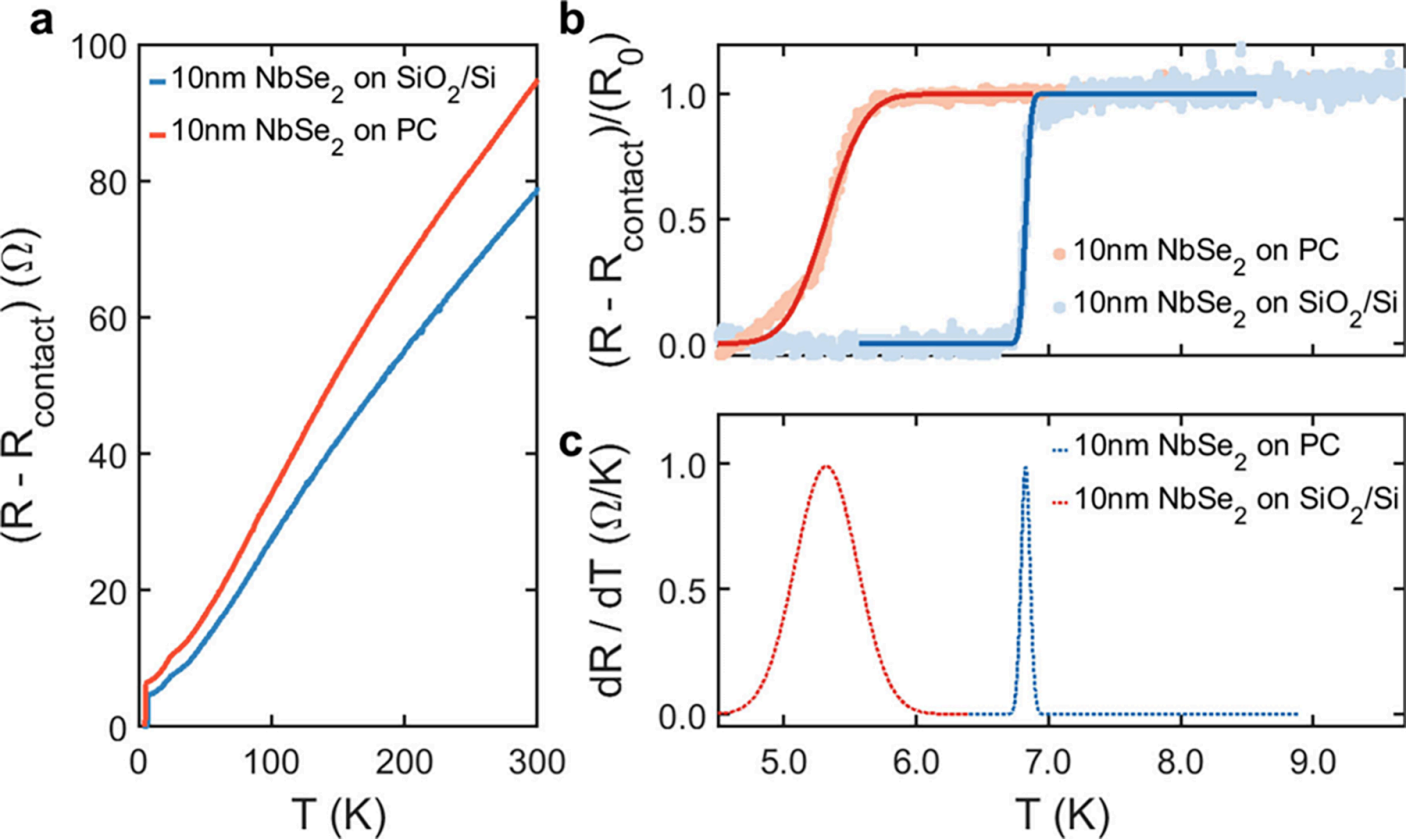
(a) Temperature dependence of two-terminal resistance for 10 nm thick NbSe_2_ devices on SiO_2_/Si and PC substrates, respectively. Contact resistance (*R*_contact_) has been subtracted for comparison purposes (see the Materials and Methods section). The blue (red) lines correspond to the device shown in Figure 1c (Figure 1d). Comparison of critical temperature for both devices: (b) A zoomed-in view from panel (a) around the superconducting transition temperature. Dots represent experimental data. Solid lines represent a step function fit of the data points. Curves have been normalized by the height (*R*_0_) of the step function fit. (c) Derivative of the step function fit in panel b with respect to temperature (d*R*/d*T*), emphasizing the difference in position and width of the superconducting transition between the two devices.

To accurately assess the differences in critical temperature and the width of the phase transition between samples, we fitted the experimental data points to a broadened step function (Figure 2b) of the form , where *erf* is the error function, *T*_c_ is the position of the step, that we identify with the critical temperature for the superconducting transition. *R*_*contact*_ is an offset representing the contact resistance, or residual resistance, while *R*_0_ and Δ are the height and the width of the step, representing respectively the resistance of the normal state and the broadening of the transition (see Supporting Information, Section S2). For convenience, the experimental data are normalized by the height (*R*_0_) of the step function in Figure 2b. The numerical derivative of the fitting result - a Gaussian centered at *T*_c_ with full width at half-maximum (fwhm) equal to Δ - is presented in Figure 2c for a graphical comparison of the phase transition critical temperature *T*_c_ and its broadening (Δ) for both devices. From this analysis, it is extracted that the 10 nm thick NbSe_2_ device on SiO_2_/Si displays a sharper (Δ = 79 mK) superconducting transition at *T*_c_ = 6.8 K, while its PC device counterpart exhibits a broader (Δ = 550 mK) superconducting transition at *T*_c_ = 5.3 K, representing a significant decrease of approximately 1.5 K and a widening of 476 mK for the NbSe_2_ on PC compared to the device on SiO_2_/Si.

Polycarbonate possesses a 2 orders of magnitude higher thermal expansion coefficient (α ∼ 6.5 × 10^–5^ K^–1^)^20^ than SiO_2_/Si (α ∼ 5 × 10^–7^ K^–1^).^29^ This difference is expected to persist at low temperatures. Since the strain effects due to the compression of SiO_2_/Si are negligible, SiO_2_/Si-based devices are excellent control samples for isolating the effects of the induced strain by the thermal compression of polycarbonate. Both NbSe_2_ devices have similar thickness and were mounted and measured simultaneously close to the cold plate of a cryostat. Therefore, the observed modulation in the critical temperature on the PC device, compared to the SiO_2_/Si device, must arise from the induced biaxial compressive strain induced by the thermal compression of PC during the cooling down from 300 to 4.3 K.

According to refs.,^8,18^ applying biaxial strain to 2D Bardeen-Cooper-Schrieffer (BCS) superconductors can generate significant changes in the density of states (DOS) at the Fermi level, which, together with the strain-induced changes in phonon modes, result in a modulation of the electron–phonon coupling strength leading to variations in the critical temperature of their superconducting transition. Specifically, biaxial compressive strain is expected to weaken electron–phonon coupling, due possibly to a decrease of the DOS at the Fermi level and to the increase of the stiffness of the in-plane lattice vibrations, resulting in a reduction of *T*_c_. Furthermore, in multilayer samples the reduction of the in-plane lattice constants is accompanied by an increase of the interlayer distance, because of the Poisson’s effect,^30,31^ further contributing to changes in the electron–phonon coupling. Therefore, the increase in the interlayer distance in multilayer samples under compressive biaxial strain likely favors a behavior that resembles that of single-layers, explaining the observed decrease of *T*_c_ in our samples and contrary to the increase of *T*_c_ observed in the applied pressure experiments.

The results extracted for the 10 nm thick samples already suggest that biaxial compressive strain can be effectively transferred from the substrate, up to a large number of layers (∼14), in spite of the weakness of the van der Waals interactions between them. The increased width of the NbSe_2_ superconducting transition on PC compared to SiO_2_/Si may arise from the expected variations of compression levels across the out-of-plane direction in the sample. To further investigate the extent of this result to samples with a larger number of layers, we deposited another seven NbSe_2_ flakes with various thickness ranging from 12 to 86 nm onto PC substrates with prepatterned contacts, similar to that in Figure 1a,c. For each of those devices, we prepared another flake of similar thickness on SiO_2_/Si, which was measured simultaneously to its PC counterpart. This approach enabled us to isolate the effects of the induced strain transferred from the PC to the material, discarding other sources of changes in the critical temperature, such as thickness variations.

Figure 3a presents the temperature dependence of two-terminal resistance around the superconducting transition for all measured NbSe_2_ flakes–with thickness ranging from 10 to 86 nm–deposited onto prepatterned electrodes on PC and SiO_2_/Si substrates, respectively. A substantial decrease in the transition temperature and a broadening of the superconducting transition are observed for all PC flakes compared to those on SiO_2_/Si. To quantify this observation, experimental data were fitted to a broadened step function to extract the critical temperature position and transition width for each flake (see Table S1 for a summary of the fit parameters for all SiO_2_/Si and PC devices).

**Figure 3:**
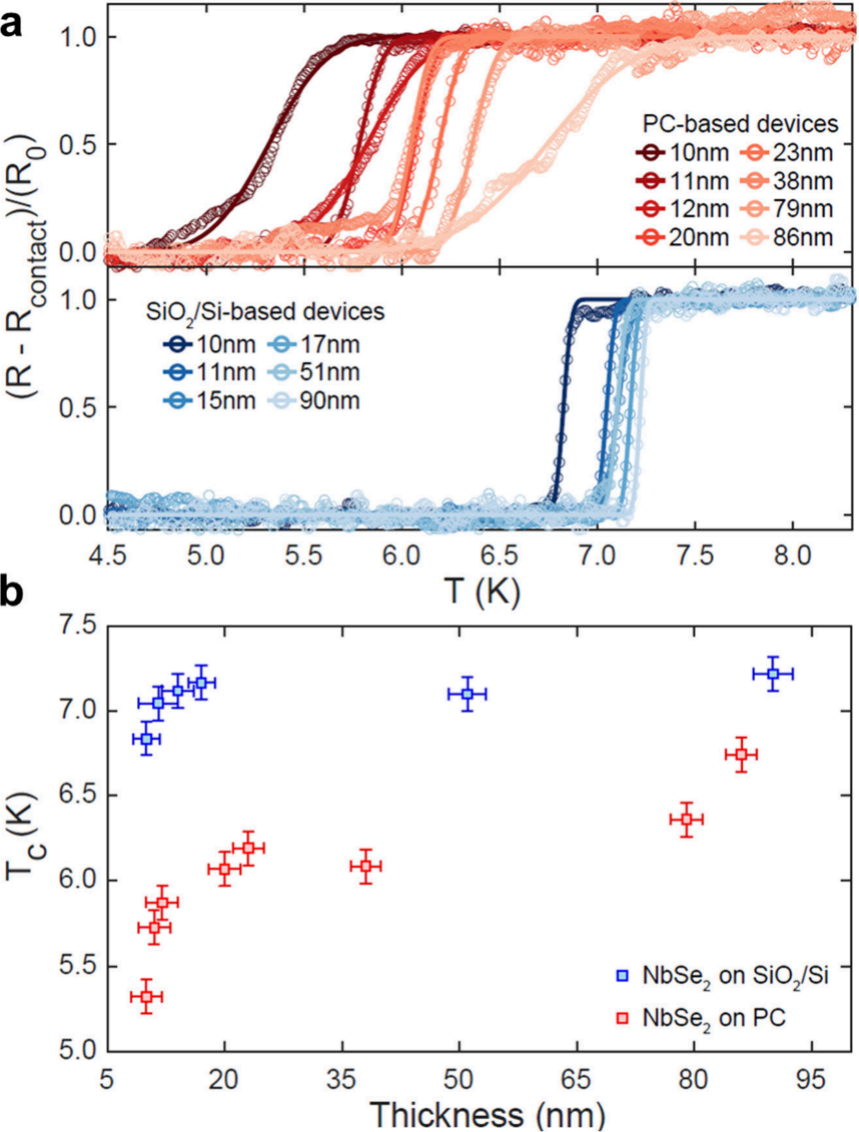
(a) Resistance as a function of temperature around the superconducting transition temperature for NbSe_2_ devices. Top: PC-based devices prepared from NbSe_2_ flakes with thickness ranging from 10 to 86 nm. Bottom: SiO_2_/Si-based devices prepared from NbSe_2_ flakes with thickness ranging from 10 to 90 nm. Solid lines represent the fit of experimental data points to a broadened step function. The residual resistance (*R*_contact_) has been subtracted and the curves have been normalized by the height (*R*_0_) of the step function fit. (b) Transition temperature (*T*_c_) extracted from the step-function fits, shown in panel (a), for SiO_2_/Si-based devices (blue markers) and PC-based devices (red markers), respectively, as a function of flake thickness. Vertical error bars correspond to the uncertainty in the measurement of temperature (±0.1 K), while thickness error is obtained from the AFM height determination (±2 nm).

The values for the superconducting critical temperature *T*_c_ extracted for all devices with flakes of different thicknesses are summarized in Figure 3b. Even though superconductivity is usually decreased in few-layered films due to disorder-induced localization of Cooper pairs and other phenomena affecting the superconducting energy gap and the electronic band structure,^26^ all devices fabricated on PC are thick enough (>13 layers) to consider that the observed drop on *T*_c_ cannot simply stem from their reduced thickness. Furthermore, for all thickness values, the critical temperature of NbSe_2_ devices fabricated on PC is systematically lower than that of similar devices on SiO_2_/Si. Electrostatic tuning has been demonstrated to modulate the *T*_c_ of ultrathin NbSe_2_ flakes (2–3 layers) by up to 0.2 K.^10^ However, this effect is significantly weaker in relatively thick flakes (∼10 nm) due to the material’s metallic character, leading to *T*_c_ changes of only tens of mK.^33^ Therefore, the differences in electrostatic effects between samples on SiO_2_/Si and PC substrates cannot explain the observed variations in *T*_c_. Thus, the changes in *T*_c_, even for our thicker devices, must stem from the strain induced by the thermal compression of the PC substrates.

Notably, the strain-induced modulation in *T*_c_ is larger for thinner samples and decreases with increasing the number of NbSe_2_ layers. This change in *T*_c_ is also accompanied by an overall broadening of the transition for PC samples compared to SiO_2_/Si ones. However, while a clear correlation appears between *T*_c_ and flake thickness, the specific values of transition width seem to be sample-dependent (for details see Supporting Information, Sections S3 and S4).

Interestingly, the effects of strain remain noticeable in samples with thickness exceeding 80 nm. This indicates that the biaxial compressive strain transferred from the substrate to the sample can significantly affect the physical phenomena governing superconductivity, even in samples with thicknesses close to the bulk limit. Therefore, our results suggest an efficient strain transfer between van der Waals layers. In principle, strain transfer between layers is expected to be poor due to the weakness of van der Waals interactions compared to covalent bonds.^34^ In such a scenario, even if the transfer of strain from the substrate to the first layer were perfect, the level of transferred strain would be expected to decay quickly across the very few layers nearest to the substrate. In other words, the effects of the strain induced from the substrate would be significant only for the few closest layers, with most of the material remaining unaffected in samples with larger thickness. In contrast, our results demonstrate that strain can be efficiently transferred across several van der Waals layers. Even though the effects of strain seem to decrease as the number of layers increases, strain transfer remains efficient enough to significantly impact superconductivity in samples close to the bulk limit. The fact that the broadening does not show a clear thickness dependence suggests a uniform strain transfer in the out-of-plane direction for both thinner and thicker flakes. The increasing role of van der Waals interlayer interactions in thicker samples seem to affect the total degree of strain induced in the sample.

In conclusion, we investigated the effects of large and uniform biaxial compressive strain on the superconducting phase transition of 2H-NbSe_2_ devices consisting of flakes with thickness ranging from 10 to 86 nm deposited onto polycarbonate substrates. The induced strain, which arises from the large thermal compression (>1.2%) of the polycarbonate substrates as they are cooled from room temperature down to 4.3 K, remarkably modulates the critical temperature of the NbSe_2_ superconducting phase (by up to 1.5 K). This modulation of critical temperature decreases with increasing flake thickness. However, *T*_c_ modulations induced by the biaxial compressive strain transferred from the substrate remain significant in samples up to 80 nm. Thus, our findings indicate that strain can be efficiently transferred between van der Waals layers, suggesting that the strain induced by substrate deformation can have substantial effects in rather thick films of 2D materials, contrary to previous theoretical predictions. In summary, our results provide experimental evidence of how biaxial compressive strain can be efficiently transferred from a polymeric substrate to multilayer 2D material samples, offering an affordable method to tune phase transitions and other quantum properties at very low temperatures.

## Materials and Methods

### Fabrication and Characterization of NbSe_2_ samples

A 2H-NbSe_2_ crystal (HQ Graphene) was mechanically exfoliated using 1008 silicon-free blue tape (Medium-High Tack type from Ultron Systems Inc.) onto a transparent polydimethylsiloxane (PDMS) (Gel-Film WF 4x 6.0 mil from Gel-Pak) substrate for inspection under a modified optical metallurgical microscope. The exfoliation and inspection were carried out under ambient conditions. The ratio between the measured transmitted light at sample (NbSe_2_) and substrate (Gel-Film), acquired using a white light source and a modified Motic BA310 microscope following ref.,^35^ yields relative transmittance spectra (see Supporting Information, Section S1 for details). By modeling the multilayer system, using the real and complex components of the refractive index^36^ of bulk NbSe_2_, we were able to estimate the thickness of the flakes from their relative transmittance. Selected flakes were transferred by a dry transfer method^19^ either onto a 250 μm thick polycarbonate film with size of 3 × 6 mm^2^ with prepatterned Ti/Au (5 nm/45 nm) electrodes or a 3 × 6 mm^2^ Si/SiO_2_ substrate with a 300 nm oxide layer with prepatterned Ti/Au (5 nm/50 nm) electrodes. In both cases, electrodes were prepared by electron beam evaporation through a metal shadow mask acquired from Ossila. Flake thickness was corroborated by AFM topography after electrical measurements were finalized.

### AFM Measurements

Atomic Force Microscopy (AFM) measurements were performed using an NTEGRA SPECTRA AFM setup from NT-MDT Spectrum Instruments Company, with AC-160 cantilevers in AFM in amplitude modulation noncontact mode. The AFM topographic images were obtained at a resolution of 512 × 512 pixels with a frequency of 0.1 Hz. Image treatment was performed using Gwydion Software: plane and line to line corrections were performed to ensure an accurate determination of height.

### Low-Temperature Transport Measurements

Pairs of NbSe_2_ devices of similar thickness fabricated on PC and on SiO_2_/Si substrates were loaded together and simultaneously measured in a closed-cycle cryostat (AttoDry 800, Attocube Gmbh), allowing to cool down to a base temperature of 4.2 K under cryogenic vacuum conditions (<10^–6^ mbar). Temperature was ramped at a rate of ∼1 K/min. Sample temperature was measured by a commercial thermometer (DT-670 silicon diode from Lakeshore Cryotronics) clamped to the same plate as the NbSe_2_ devices. We performed resistance measurements by using two lock-in amplifiers (Stanford Research System, SR830). Two terminal resistance measurements were performed in constant current mode, with amplitude 25 nA and frequency 293 or 307 Hz.

## Supporting Information Available

Information on the optical characterization of NbSe_2_ flakes and further details on resistance data analysis, such as fitting parameters and transition width as a function of thickness (PDF)
