# Supplementary material for: Modulation of the Superconducting Phase Transition in Multilayer 2H-NbSe2 Induced by Uniform Biaxial Compressive Strain
Source: Nano Lett. 2024 Jul 30;24(34):10504–9. doi: 10.1021/acs.nanolett.4c02421 (PMC11363131; doi:10.1021/acs.nanolett.4c02421)
Supplement: Supplementary file 1 — nl4c02421_si_001.pdf [file nl4c02421_si_001.pdf]

Supporting Information for

Modulation of the Superconducting Phase Transition in Multilayer 2H-NbSe$_2$ induced by Uniform Biaxial Compressive Strain

Eudomar Henríquez-Guerra [1,2,3], Lisa Almonte [1,2,3], Hao Li [4,+], Daniel Elvira [1,2], M. Reyes Calvo [1,2,3,5,], Andres Castellanos-Gomez [4,*]*

[1] Departamento de Física Aplicada, Universidad de Alicante, 03690 Alicante, Spain

[2] Instituto Universitario de Materiales IUMA, Universidad de Alicante, 03690, Alicante, Spain

[3] BCMaterials, Basque Center for Materials, Applications and Nanostructures, 48940 Leioa, Spain

[4] 2D Foundry group, Instituto de Ciencia de Materiales de Madrid, Consejo Superior de Investigaciones Científicas, 28049, Madrid, Spain

[5] IKERBASQUE, Basque Foundation for Science, Plaza Euskadi 5, 48009 Bilbao, Spain

[+]Current address: The Institute of Physics, Chinese Academy of Sciences P.O.Box 603, 100190, Beijing, China.

*E-mail: reyes.calvo@ua.es, andres.castellanos@csic.es

Contents

S1. Optical characterization of NbSe$_2$ devices

S2. Parameters from fitting two-terminal resistance data to a broadened step function

S3. Two-terminal resistance analysis for all NbSe$_2$ devices

S4. Transition width versus flake thickness

S1. Optical characterization of $NbSe_2$ devices

The $NbSe_2$ flakes were mechanically exfoliated onto a transparent polydimethylsiloxane substrate (gel film from Gel-Pak®) for examination under an optical microscope (Motic BA310 Met-T). Images of the flakes on Gel-Film in transmission and reflection mode were captured using the Motic BA310 microscope fitted with a digital CMOS Camera (AMScope MU1803).

Figures S1a (**S1b**) and **S2a** (**S2b**) present the optical image in reflection (transmission) mode of the 10 nm thick $NbSe_2$ flakes presented in Figure 1 of the main text, which were eventually deposited onto a Si/SiO_2 and a polycarbonate (PC) substrate, respectively.

To obtain the relative transmittance spectrum of the $NbSe_2$ flakes, the samples were illuminated with a white light source positioned beneath them. The Motic BA310 microscope was modified to perform spectroscopy measurements following Ref. [s1]. Light from a spot measuring a few micrometers (~3 µm) in diameter at the center of the sample was collected and guided through an optical fiber (105 µm in diameter) to a CCS200/M compact spectrometer (Thorlabs). The acquired spectra from the sample and from the substrate are used to calculate the relative transmittance (RT) as $RT = (I_{\text{sample}})/I_{\text{substrate}}$, where I_{sample} and $I_{\text{substrate}}$ are the transmitted light intensities at the sample and substrate, respectively.

The relative transmittance spectrum can be simulated using the transfer matrix method, which applies Fresnel equations for light propagation in optical multilayers to model the intensity of the light beam transmitted with normal incidence across the sample [**s2**]. This approach yields an estimation of the layers thickness if their refractive index is known. In this model, we utilized the available refractive index data for bulk $NbSe_2$ from Ref. [**s3**] and considered each flake sandwiched between two semi-infinite media: air and Gel-Film, with real refractive indices of $n_{air} = 1$ and $n_{PDMS} = 1.43$, respectively. These values are considered to be constant for the range of experimental wavelengths. It's important to note that the refractive index value for the Gel-Film substrate has been chosen to match that of polydimethylsiloxane (PDMS) [**s4**].

Figures **S1c** and **S2c** present the relative transmittance spectrum at the center of the two 10 nm thick $NbSe_2$ flakes, which are presented in the Figure 1 of the main text after being deposited onto a Si/SiO_2 and a polycarbonate (PC) substrate, respectively.

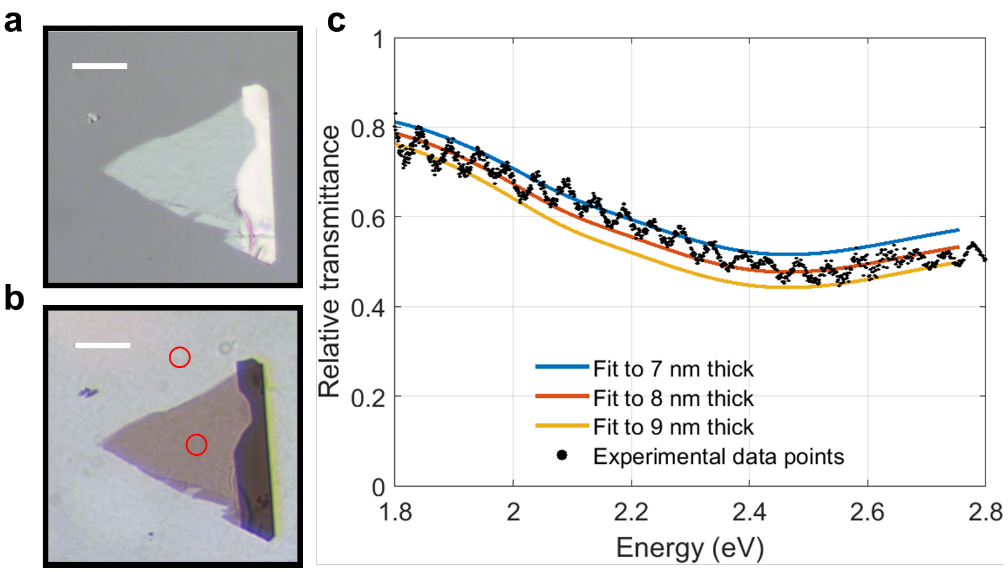

Figure S1: Optical image in (a) reflection and (b) transmission mode of the 10 nm thick $NbSe_2$ on Gel-film substrate. This same flake is presented in Figure 1c of the main text after being deposited on Si/SiO_2. (c) Relative transmittance spectrum obtained from the spectra acquired at the flake and gel-film substrate spots marked (red circles) in panel b. The solid lines correspond to the simulated results from the transfer-matrix model.

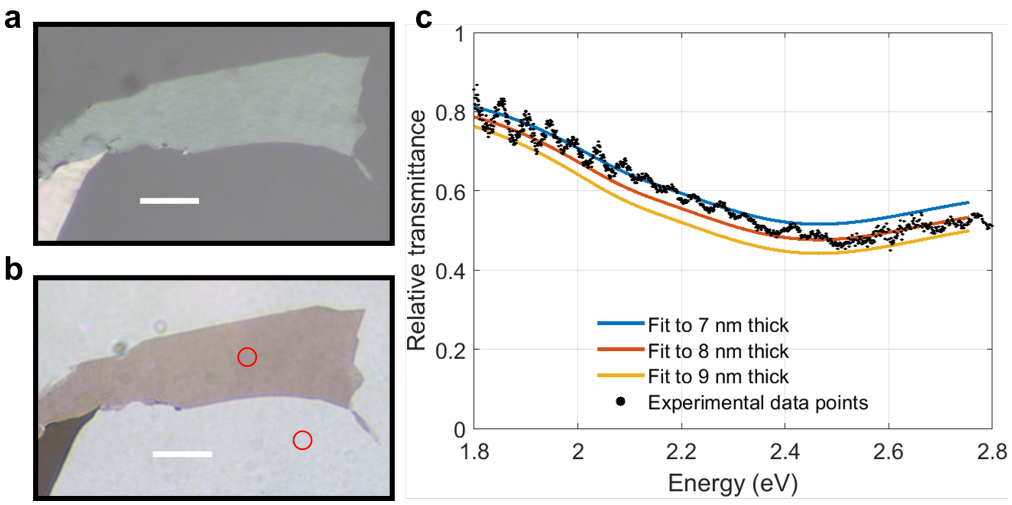

Figure S2: Optical image in (a) reflection and (b) transmission mode of the 10 nm thick $NbSe_2$ flake on Gel-film substrate. This same flake is presented in Figure 1d of the main text after being deposited on a PC substrate. (c) Relative transmittance spectrum obtained from the spectra acquired at the sample and gel-film substrate spots marked (red circles) in panel b. The solid lines correspond to the simulated results from the transfer-matrix model.

S2. Parameters from fitting two-terminal resistance data to a broadened step function

Table S1. Fitting parameters of critical temperature (T_c), broadening (Δ), height (R_0) and residual resistance ($R_{contact}$) of the superconducting phase transition to a broadened step function. Error is estimated from 95% confidence bounds of the fit.

**$NbSe_2$ flakes deposited onto a polycarbonate (PC) substrate**				
Thickness (nm)	$R_{contact}$ (Ω)	R_0 (Ω)	T_c (K)	Δ (mK)
10 ± 2	28.36 ± 0.06	6.16 ± 0.07	5.32 ± 0.01	550 ± 20
11 ± 2	29.73 ± 0.03	2.19 ± 0.04	5.78 ± 0.01	230 ± 30
12 ± 2	16.81 ± 0.03	4.10 ± 0.04	5.82 ± 0.01	570 ± 20
20 ± 2	29.56 ± 0.01	1.15 ± 0.02	6.07 ± 0.01	200 ± 30
23 ± 2	16.23 ± 0.02	1.00 ± 0.03	6.19 ± 0.01	210 ± 50
38 ± 2	30.17 ± 0.02	0.60 ± 0.03	6.05 ± 0.02	180 ± 50
79 ± 2	26.51 ± 0.01	0.34 ± 0.02	6.36 ± 0.03	230 ± 100
86 ± 2	27.07 ± 0.02	0.38 ± 0.03	6.74 ± 0.07	800 ± 200
**$NbSe_2$ flakes deposited onto a Si/SiO_2 substrate**				
Thickness (nm)	$R_{contact}$ (Ω)	R_0 (Ω)	T_c (K)	Δ (mK)
10 ± 2	28.02 ± 0.02	4.53 ± 0.04	6.826 ± 0.003	77 ± 9
11 ± 2	32.13 ± 0.02	9.15 ± 0.03	7.048 ± 0.001	79 ± 4
15 ± 2	27.86 ± 0.02	4.35 ± 0.03	7.105 ± 0.003	128 ± 9
17 ± 2	21.48 ± 0.01	2.93 ± 0.02	7.169 ± 0.002	70 ± 8
51 ± 2	30.65 ± 0.01	0.87 ± 0.02	7.101 ± 0.007	85 ± 24
90 ± 2	27.33 ± 0.01	0.94 ± 0.02	7.219 ± 0.005	58 ± 16

S3. Two-terminal resistance analysis for all $NbSe_2$ devices

A. PC-based devices

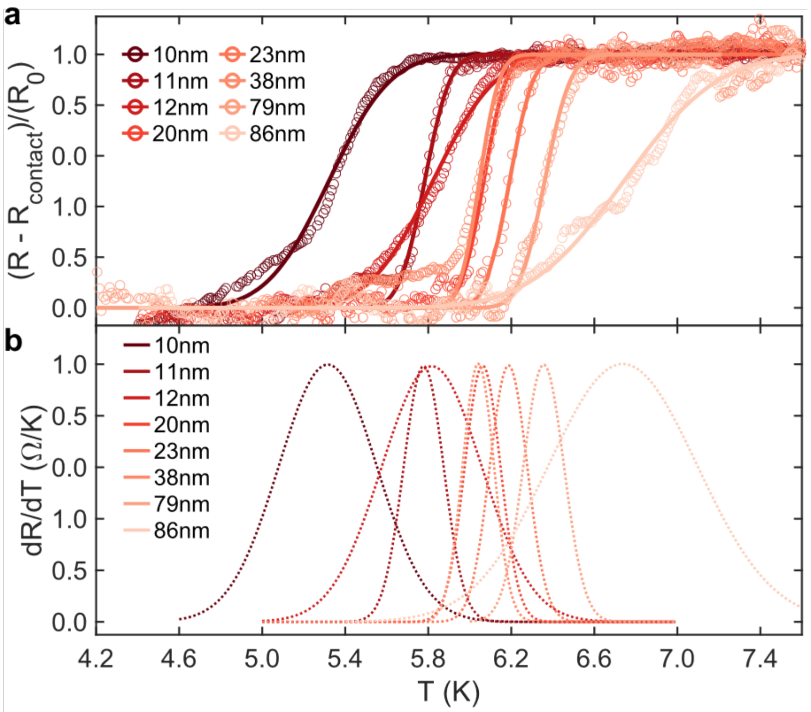

Figure S3: Graphical comparison of critical temperature and transition width for all PC-based devices with thickness as indicated in the legend: (a) A zoomed-in view of the two-terminal resistance around the superconducting transition temperature. Dots represent experimental data. Solid lines represent a step function fit of the data points. Curves have been normalized by the height (R_0) of the step function fit. (b) Derivative of the step function fit with respect to temperature (*dR/dT*), emphasizing a notable difference in superconducting transition between all devices, primarily attributed to the induced strain transferred from the PC substrate.

B. SiO_2/Si-based devices

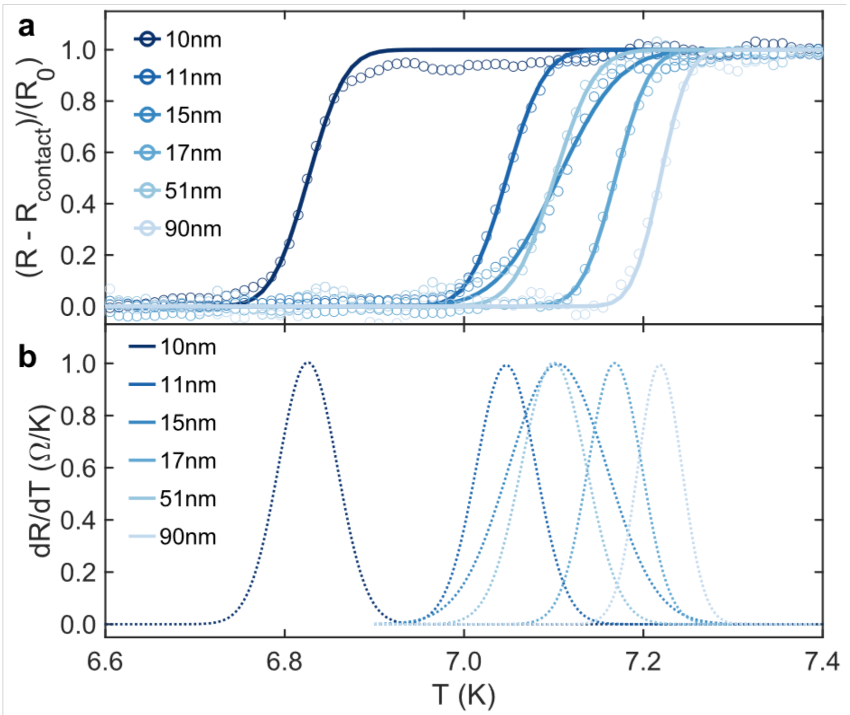

Figure S4: Graphical comparison of critical temperature and transition width for all Si/SiO_2-based devices with thickness as indicated in the legend: (a) A zoomed-in view of the two-terminal resistance around the superconducting transition temperature. Dots represent experimental data. Solid lines represent a step function fit of the data points. Curves have been normalized by the height (R_0) of the step function fit. (b) Derivative of the step function fit with respect to temperature (dR/dT), emphasizing a notable small difference in superconducting transition between all devices, mainly attributed to changes in thickness.

S4. Transition width versus flake thickness

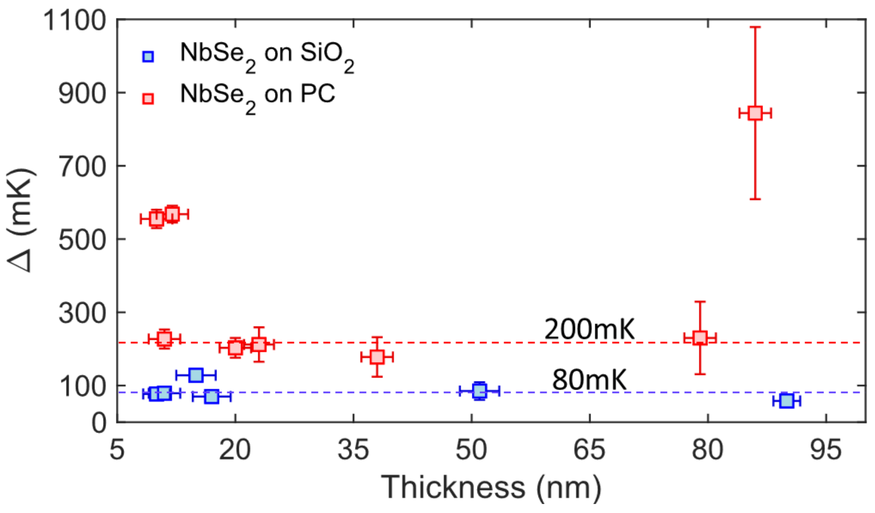

Figure S5: Thickness-dependent evolution of the broadening (Δ) of the superconducting phase transition, extracted from the step-function fits applied to resistance experimental data points for Si/SiO_2-based devices (blue markers) and PC-based devices (red markers). The vertical error bars correspond to the statistical uncertainty of the fit, while the horizontal error bars represent the AFM uncertainty of ±2 nm.

Supplementary references

[s1] Frisenda, R.; Niu, Y.; Gant, P.; Molina-Mendoza, A. J.; Schmidt, R.; Bratschitsch, R.; Liu, J.; Fu, L.; Dumcenco, D.; Kis, A.; Lara, D. P. D.; Castellanos-Gomez, A. Micro-Reflectance and Transmittance Spectroscopy: A Versatile and Powerful Tool to Characterize 2D Materials. J. Phys. D: Appl. Phys. 2017, 50 (7), 074002. https://doi.org/10.1088/1361-6463/aa5256.

[s2] Born, M. & Wolf, E. Principles of Optics: Electromagnetic Theory Of Propagation, Interference and Diffraction of Light. (Pergamon, Oxford; New York, 1980).

[s3] Munkhbat, B.; Wróbel, P.; Antosiewicz, T. J.; Shegai, T. O. Optical Constants of Several Multilayer Transition Metal Dichalcogenides Measured by Spectroscopic Ellipsometry in the 300–1700 Nm Range: High Index, Anisotropy, and Hyperbolicity. ACS Photonics 2022, 9 (7), 2398–2407. https://doi.org/10.1021/acsphotonics.2c00433.

[s4] Cardenas-Valencia, A. M.; Dlutowski, J.; Fries, D.; Langebrake, L. Spectrometric Determination of the Refractive Index of Optical Wave Guiding Materials Used in Lab-On-a-Chip Applications. Appl. Spectrosc., AS 2006, 60 (3), 322–329.
